# Supplementary material for: Mapping CircRNA–miRNA–mRNA regulatory axis identifies hsa_circ_0080942 and hsa_circ_0080135 as a potential theranostic agents for SARS-CoV-2 infection
Source: PLoS One. 2023 Apr 13;18(4):e0283589. doi: 10.1371/journal.pone.0283589 (PMC10101458; doi:10.1371/journal.pone.0283589)
Supplement: Attachment — Submitted filename: Manuscript Number PONE D 22 34657.docx [file pone.0283589.s001.docx]

Manuscript Number: PONE-D-22-34657

Title: “Mapping CircRNA–miRNA–mRNA Regulatory Axis Identifies hsa_circ_0080942 and hsa_circ_0080135 as a potential theranostic agents for SARS-CoV-2 infection”

This is an interesting small study and the authors have collected a unique dataset using cutting edge methodology. The paper is well written and structured.

The following minor issues should be addressed:

1)    In introduction section first paragraph author may update number of total people have been infected and death globally with virus SARS-CoV-2 with updated references.
